# Screening of active components of melastoma dodecandrum lour. against diabetic osteoporosis using cell membrane chromatography-mass spectrometry

**DOI:** 10.3389/fphar.2024.1450154

**Published:** 2024-10-25

**Authors:** Xiaoqin Zhang, Jiale Mao, Lu Shao, Shuang Liu, Jiwang Zhou, Mingrong Mei, Zunjing Zhang

**Affiliations:** ^1^ Pharmacy Department, Lishui TCM Hospital Affiliated to Zhejiang Chinese Medical University, Lishui Hospital of Traditional Chinese Medicine, Lishui, China; ^2^ Zhejiang Provincial Key Laboratory of She Medicine Inheritance, Innovation, Development and Application of Traditional Chinese Medicine, Lishui, China; ^3^ Lishui She Medicine Inheritance, Innovation, Development and Application Key Laboratory of Traditional Chinese Medicine, Lishui, China; ^4^ School of Pharmaceutical Sciences, Zhejiang Chinese Medical University, Hangzhou, China; ^5^ Qingdao Hiser Hospital Affiliated of Qingdao University, Qingdao Traditional Chinese Medicine Hospital, Qingdao, China; ^6^ Zhejiang Provincial Ethnic Hospital, Jingning, China

**Keywords:** melastoma dodecandrum lour., isovitexin, RAGE pathway, diabetic osteoporosis, cell membrane chromatography

## Abstract

**Background:**

Melastoma dodecandrum Lour. (MD), a traditional botanical drug known for its hypoglycemic, antioxidant, and anti-inflammatory properties, is commonly used to treat diabetes, osteoarthritis, and osteoporosis. However, its specific active components against diabetic osteoporosis remain unclear.

**Purpose:**

This study aimed to identify the key active components in MD using cell membrane chromatography coupled with mass spectrometry and validate their effects *in vitro*.

**Methods:**

An AGEs-induced osteoblast injury model was established. MTT assays measured cell viability, and ALP activity was assessed using a biochemical kit. Western blotting was employed to detect the expression levels of osteoblast-related proteins OCN and RUNX2 and the AGE receptor protein RAGE. ELISA was used to determine the levels of SOD, MDA, CAT, and GPx. PCR quantified TNF-α expression to evaluate the protective effects and potential mechanisms of MD. The AGEs-induced osteoblast cell membrane chromatography-mass spectrometry method facilitated the rapid identification of potentially active compounds based on their affinity for the osteoblast cell membrane. Cell experiments further validated the activity of the characteristic component isovitexin.

**Results:**

MD significantly improved cell viability in AGEs-damaged osteoblasts, enhanced ALP, SOD, CAT, and GPx activities, reduced MDA levels, increased OCN and RUNX2 protein expression, and decreased TNF-α mRNA and RAGE protein expression. Cell membrane chromatography identified 20 chemical constituents, including 13 flavonoids, 4 organic acids, 1 phenylpropanoids, 1 terpenoids, and 1 alkaloid. Cell experiments have confirmed that isovitexin has significant protective activity against osteoblasts and can inhibit the expression of specific receptor RAGE on the osteoblast membrane, consistent with the effect of MD.

**Conclusion:**

MD and its active component, isovitexin, provide protective effects against AGEs-induced osteoblast injury, offering a basis for subsequent drug development.

## 1 Introduction

Melastoma dodecandrum Lour. (MD) belongs to the Melastomataceae family and is used in fresh and dried forms. It is known for its heat-clearing, detoxifying, blood-cooling, hemostatic, yin-nourishing, and dryness-reducing properties. This plant is widely distributed in Zhejiang, Guangdong, Guangxi, and Guizhou provinces of China (Wang J et al., 2017). The She ethnic group has traditionally used MD for its medicinal properties. Modern research indicates that MD contains various chemical components such as fatty acids, organic acids, polysaccharides, essential oils, flavonoids, tannins, triterpenes, and steroids. These components exhibit significant pharmacological effects, including hypoglycemic activity, improvement of insulin resistance, anti-inflammatory effects, and inhibition of oxidative stress (Huang et al., 2021; Xu et al., 2023). Studies have reported that MD extracts can treat type 2 diabetes in rats by regulating lipid metabolism disorders (Weng et al., 2019). Yang et al. discovered that MD extracts exhibit strong α-glucosidase inhibitory activity, significantly reducing postprandial blood glucose levels in maltose-fed mice. Xu (2023) found that 50% ethanol extracts of MD promote osteoblast differentiation, inhibit osteoclast formation, and treat osteoporosis in ovariectomized mice. However, the efficacy and active components of MD in treating diabetic osteoporosis (DOP) remain unclear.

DOP is a degenerative, systemic, and metabolic bone disease induced by diabetes, characterized by reduced bone mass, increased bone fragility, and a higher risk of fractures (Mohsin et al., 2019). It is a major chronic complication of diabetes in the skeletal system, significantly complicating treatment and increasing the economic burden on patients. Research indicated that the accumulation of advanced glycation endproducts (AGEs) is a key factor in the development of DOP (Wang et al., 2020; Cheng et al., 2018). AGEs inhibit osteoblast differentiation and promote apoptosis, thereby contributing to osteoporosis (Li et al., 2020; Li et al., 2021; Weinberg et al., 2014; Suh et al., 2014; Tanaka et al., 2013; Ge et al., 2022). AGEs are highly active end products formed by non-enzymatic glycation reactions (the Maillard reaction) between the amino groups of proteins, fatty acids, or nucleic acids and the aldehyde groups of reducing sugars. Under physiological conditions, AGEs are maintained at low levels in the body, but their formation increases under pathological conditions such as diabetes and aging (Takeuchi et al., 1999; Lin et al., 2018). AGEs exert biological effects primarily by binding to the receptor for advanced glycation endproducts (RAGE) on cell surfaces, participating in intracellular signal transduction, and stimulating the release of cytokines. The binding of AGEs to RAGE receptors on osteoblasts, osteocytes, and osteoclasts impairs their functions, promoting the development of DOP.

Traditional Chinese Medicine (TCM) is a unique and valuable resource in China, serving as a treasure trove of natural lead compounds. TCM plays a significant role in the prevention and treatment of various human diseases due to its remarkable efficacy and minimal side effects, gaining global attention and recognition. However, the complex chemical composition of TCM presents a challenge for the precise identification of its active components, which is crucial for understanding its pharmacological basis. Cell membrane chromatography (CMC) is an advanced chromatographic technique that employs cell membranes containing target receptors as the stationary phase. It leverages the specific recognition and affinity between drugs and membrane receptors to rapidly screen active components from complex TCM systems. CMC is characterized by its high efficiency, sensitivity, and automation, making it suitable for screening active components in TCM. This technology, based on multi-component-target affinity interactions, has been widely applied in the research of TCM (Han et al., 2018). The authors of this study have previously established a stable CMC screening system based on osteoblasts (Wang N et al., 2017) and applied it to screen active components in various TCM materials and preparations, such as Ligustrum lucidum and (Chen et al., 2022) and Epimedium (Wang N et al., 2017).

This study used AGEs to induce osteoblasts to a pathological state, and a CMC-HPLC-TOF-MS method was established. This method employs the affinity between compounds and osteoblast cell membranes to rapidly identify potentially active compounds in MD. Specifically, the characteristic component isovitexin was validated for its activity. The aim is to provide a theoretical basis and effective method for the prevention and treatment of DOP.

## 2 Materials and methods

### 2.1 Description of MD extract

The MD botanical drug, Melastoma dodecandrum Lour., sourced from Lishui Hospital of Traditional Chinese Medicine (specimen number LSZ023), was approved by the unit responsible for the acceptance of Chinese medicine decoction pieces. Character identification: the root is oval, with a diameter of about 2∼3mm, and the surface is yellowish white to brownish yellow. The stem is brown, about 1.5 mm in diameter, with longitudinal stripes on the surface, fibrous roots at the nodes, and opposite leaves. The leaves are dark green, mostly shrunk and broken, and the intact ones are oval or oval after unfolding, 1–4 cm long and 0.8–3 cm wide. Only the upper edge or the lower vein has sparse strigose. Sometimes flowers or fruits can be seen, calyx 5-lobed, petals 5. Shape fruit shape spherical, the upper part flat cut, slightly constricted. Slightly sour and astringent. Processing method: Take the technical drug, remove impurities, wash, cut into sections, and dry. This approval was in accordance with the relevant provisions of the 2015 edition of the Zhejiang provincial standard for the processing of traditional Chinese medicine.

The MD botanical drug (100 g) was pulverized and subjected to reflux extraction twice using 800 mL water for 2 h as the solvent. The extract was filtered, combined, concentrated, and dried. An appropriate amount of the resulting powder was dissolved in distilled water for subsequent cell experiments.

### 2.2 Reagents

Acetonitrile and methanol (chromatographic grade) were acquired from Thermo Fisher Scientific. The following reference standards were obtained from the National Institute for Food and Drug Control (China): gallic acid (98%, batch: 110,831–201605), protocatechuic acid (98%, batch: 110,809–201906), orientin (98%, batch: 111,777–202003), luteolin (98%, batch: 11,720–201106), isovitexin (98%, batch: P15J11F118584), rutin (91.7%, batch: 100,080–201811), and chlorogenic acid (96.1%, batch: 110,753–202018). Calcitriol (97%, batch: A2119303) was purchased from Aladdin Biochemical Technology Co., Ltd. AGEs (99%, #bs-1158P) were obtained from Beijing Bioss Biotechnology Co., Ltd.

### 2.3 Cell culture and grouping

According to the method described previously (Liu et al., 2019), primary osteoblasts were prepared from the calvaria of neonatal rats and cultured in high-glucose DMEM (containing 10% fetal bovine serum and 1% antibiotics) at 37°C in a 5% CO_2_ incubator with saturated humidity. One-day-old Wistar rats were obtained from the Experimental Animal Center of Zhejiang Chinese Medical University. The experimental groups were as follows: control group, AGEs group (100 μg/mL), AGEs + calcitriol group (1 μmol/mL), AGEs + low-dose MD group (0.01 mg/ml), AGEs + high-dose MD group (0.1 mg/mL), AGEs + low-dose isovitexin group (0.1 μmol/L), and AGEs + high-dose isovitexin group (1 μmol/L). Cells in each group were treated for 24 h before proceeding to further experiments.

### 2.4 MTT assay

Primary rat osteoblasts were seeded in 96-well plates at a 5 × 10^4^/mL density and cultured at 37°C in a 5% CO2 incubator for 24 h. The culture medium was discarded, and the cells were treated according to the experimental design for 24 h, with six replicates per group. Cell viability was assessed using an MTT assay kit (#KGA312, Keygen). The optical density of each well was measured at 490 nm using a microplate reader (Multiskan GO 1510, Thermo Fisher Scientific, Massachusetts, United States).

### 2.5 ALP activity assay

When primary osteoblasts reached 80% confluence, the culture medium was discarded, and pre-warmed osteogenic induction medium (containing 10 nM dexamethasone, 10 mM β-glycerophosphate, and 50 μg/mL ascorbic acid) was added. According to the experimental design, MD, isovitexin, and calcitriol were added to the osteogenic induction medium. The medium was changed every 2–3 days, and after 7 days of culture, ALP activity was measured using an ALP assay kit (#P0321, Beyotime).

### 2.6 Western blot analysis

Total protein was extracted using a commercial kit (#KGP250, KeyGen) and quantified using a BCA protein assay kit (#KGPBCA, Keygen). Proteins were separated by electrophoresis, transferred to PVDF membranes, and blocked for 2 h. The membranes were incubated overnight at 4°C with primary antibodies against OCN (#DF12303, Affinity Biosciences, 1:2000), RUNX2 (#PB0171, Bosterbio, 1:2000), RAGE (#BM4901, Bosterbio, 1:2000), and GAPDH (#BM3874, Bosterbio, 1:2000). After washing, the membranes were incubated with goat anti-rabbit secondary antibody (1:5,000) at room temperature for 1 h. The bands were visualized using chemiluminescence (ChemiDoc XRS+, Bio-Rad, California, United States).

### 2.7 PCR

RNA was extracted using TRIzol reagent (#CW0581, Cwbio) and reverse-transcribed into cDNA using the FastKing cDNA First Strand Synthesis Kit (#KR116, Tiangen). PCR amplification was performed using TaKaRa Ex Taq^®^ (Mg^2+^ free Buffer) reagents (#RR01a.m., TaKaRa) and primers (Table 1) in a PCR instrument (T100, Bio-Rad, California, United States) under optimized conditions. The amplified products were analyzed by 2% agarose gel electrophoresis and visualized using a gel imaging system (GenoSens, 1850, Clinx, Shanghai, China). GAPDH was used as the internal reference gene.

**TABLE 1:** Primary sequence.

Gene name	Primer sequences (5′–3′)	Annealing temperature (°C)	Product size (bp)
TNF-a	Forward: TCC​AGA​ACT​CCA​GGC​GGT​GTC	57	211
	Reverse: TGG​GCT​ACG​GGC​TTG​TCA​CTC		
GAPDH	Forward: GTC​CAT​GCC​ATC​ACT​GCC​ACT​C	57	264
	Reverse: CGC​CTG​CTT​CAC​CAC​CTT​CTT​G		

### 2.8 Measurement of SOD, MDA, CAT, and GPx

Cells were seeded in 6-well plates at a density of 1×10^5^/mL. After 24 h, the culture supernatant was discarded, and the cells were treated with corresponding culture media containing different treatments. SOD, MDA, CAT, and GPx levels were measured using respective assay kits (SOD: #S0101, Beyotime; MDA: #S0131, Beyotime; CAT: #S0051, Beyotime; GPx: #S0059, Beyotime) according to the manufacturer’s instructions.

### 2.9 Preparation of standard solutions

The reference standards were accurately weighed, including gallic acid, protocatechuic acid, orientin, luteolin, isovitexin, rutin, and chlorogenic acid. The standards were dissolved in methanol to prepare stock solutions with a 1.0 mg concentration.

### 2.10 Preparation of MD sample solution

The MD botanical drug was pulverized and subjected to reflux extraction twice using water as the solvent. The extract was filtered, combined, concentrated, and dried. An appropriate amount of the resulting powder was dissolved in 50% methanol, filtered through a 0.22 μm membrane, and used for chromatographic analysis.

Following previously established methods (Wang N et al., 2017), primary rat osteoblasts in the logarithmic growth phase were treated with 100 μg/mL AGEs for 24 h, washed three times with PBS, and collected. Cells were sonicated (400 W) for 2 s, cooled for 5 s, and this cycle was repeated 20 times to lyse the cells. The mixture was centrifuged at 1,000 *g* for 10 min (4°C), and the supernatant was further centrifuged at 15,000 × g for 20 min (4°C). The residue was mixed with PBS and vortexed for 5 min to form a cell membrane suspension. Silica gel (0.05 g, 5 μm, Qingdao Makall Chemicals Co. Ltd, China) was added to the suspension and incubated overnight at 4°C. The cell membrane-silica gel mixture was washed three times with PBS, centrifuged at low temperature, and packed into stainless steel columns (10 mm × 2 mm i. d.)

### 2.11 Cell membrane chromatography

The osteoblast CMC column (10 mm × 2 mm) was connected to an LC-20AT HPLC system (SHIMADZU, Kyoto, Japan). The mobile phase consisted of ultrapure water flowing at a rate of 0.1 mL/min. A 50 μL injection volume was used, with detection carried out at a wavelength of 254 nm and the column maintained at a temperature of 37°C. The MD sample solution was injected into the column, and fractions were collected using an FRC-10A fraction collector (SHIMADZU, Kyoto, Japan) in a 32-well plate. The fractions were identified using UPLC-TOF-MS.

### 2.12 HPLC-TOF-MS conditions

HPLC was performed using a Waters ACQUITY UPLC system (Waters, United States). The chromatographic column was a DiKMA Endeavorsil C18 column (2.1 × 150 mm, 1.8 μm). The mobile phase consisted of acetonitrile (B) and 0.1% formic acid in water (A) with the following gradient elution: 0–1 min, 90% A; 1–3 min, 90%–85% A; 3–6 min, 85%–80% A; 6–10 min, 80%–75% A; 10–12 min, 75%–50% A; 12–13 min, 50%–10% A; 13–13.1 min, 10%–90% A; 13.1–15 min, 90% A. The flow rate was adjusted to 0.3 mL/min, while the column temperature was controlled at 30°C. A 3 μL injection volume was utilized for the analysis.

Mass spectrometry was conducted using a Synapt Q-TOF mass spectrometer (Waters, United States) in negative ion mode. The ion spray voltage was set to 4,500 kV, and the ion source temperature was maintained at 550°C. The de-clustering potential was set at 100 V, and the collision energy for the negative ion mode was −30 eV. The nebulizer gas and auxiliary gas pressures were both set at 50 psi. The mass scan range was m/z 50–1,000.

### 2.13 Data analysis

Data were processed and graphed utilizing GraphPad Prism 9.0 software. All data were presented as mean ± standard deviation (x ± s). One-way ANOVA was utilized for statistical analysis of multiple group comparisons. *P* < 0.05 indicated statistical significance. *P < 0.05, **P < 0.01.

## 3 Results

### 3.1 Effect of MD on the viability of AGEs-induced osteoblasts

**FIGURE 1:**
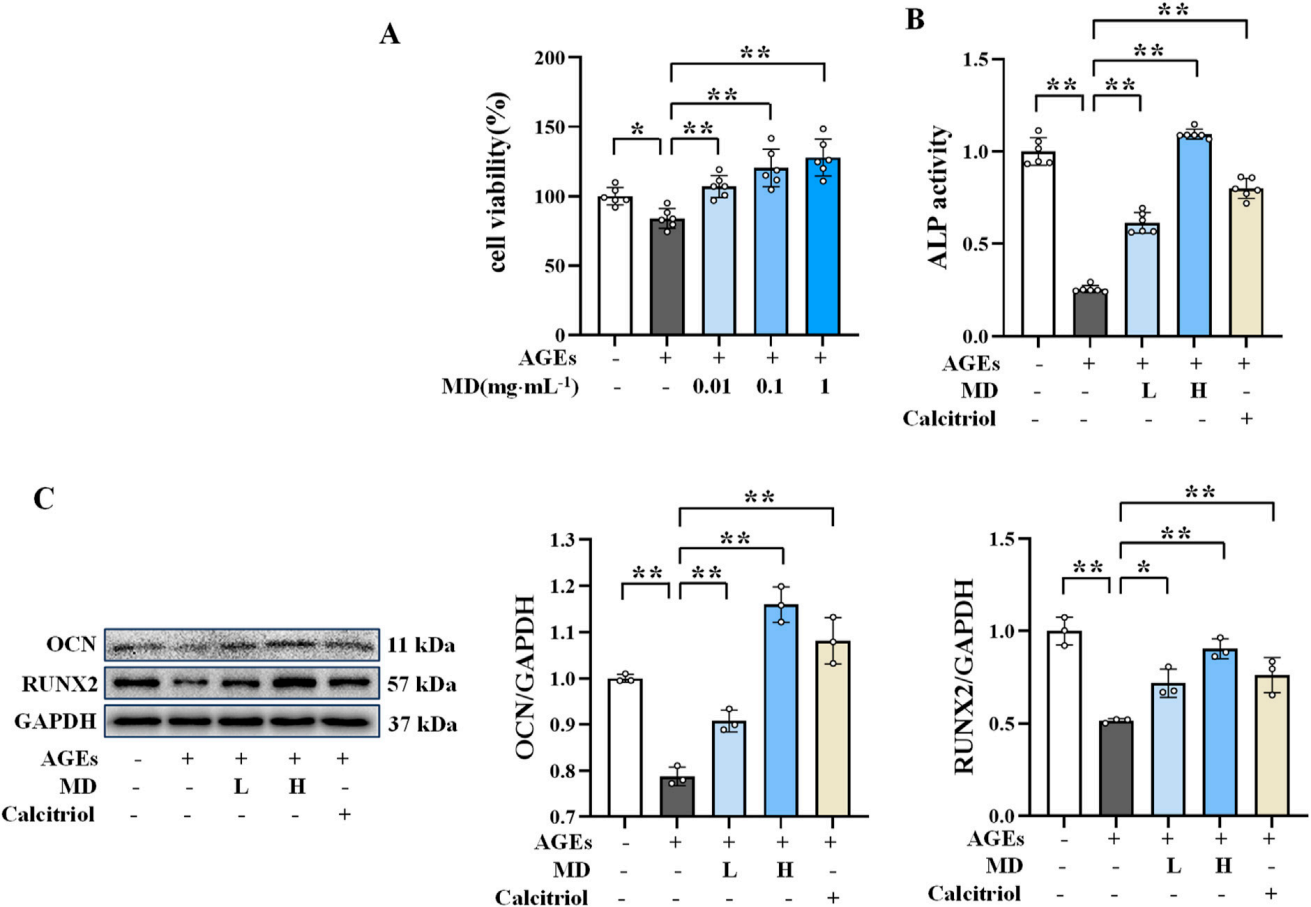
Protective effect of MD on AGEs-induced osteoblasts. **(A)** Effect of MD on the survival rate of osteoblasts (n = 6); **(B)** ALP activity in osteoblasts (n = 6); **(C)** Western blotting results of OCN and RUNX2 in osteoblasts (n = 3). *P < 0.05; **P < 0.01.

The dose-response study of MD showed that concentrations of 0.01 and 0.1 mg/mL provided significant protection against AGEs-induced osteoblast damage (Figure 1A). Osteoblasts were induced to an injured state using AGEs, followed by treatment with 0.01, 0.1, and 1 mg/mL of MD. Compared to the control group, the cell viability in the AGEs group was significantly reduced (*P* < 0.01). However, the cell viability in the MD treatment groups was significantly higher than in the AGEs group (*P* < 0.01). Both 0.01 and 0.1 mg/mL doses improved the cell viability suppressed by AGEs, and there was no significant difference in efficacy between the 0.1 and 1 mg/mL doses. Therefore, 0.01 and 0.1 mg/mL were selected as the low and high doses for further experiments.

### 3.2 Protective effect of MD on AGEs-induced osteoblasts

Considering that ALP, OCN, and RUNX2 are important markers of osteogenesis, which directly reflect the activity and function of osteoblasts (Vimalraj, 2020; An et al., 2016), the protective effect of MD on AGEs-induced osteoblasts was investigated by measuring ALP activity and the protein expression levels of OCN and RUNX2. As displayed in Figures 1B, C, the MD treatment groups and the calcitriol group exhibited significantly increased ALP activity and elevated levels of OCN and RUNX2 protein expression compared to the AGEs group (*P *< 0.01). These results indicated that both 0.01 and 0.1 mg/mL doses of MD enhance ALP activity and increase the expression levels of OCN and RUNX2 proteins, thereby protecting against AGEs-induced osteoblast damage.

### 3.3 Inhibition of AGEs-induced oxidative stress in osteoblasts by MD

**FIGURE 2:**
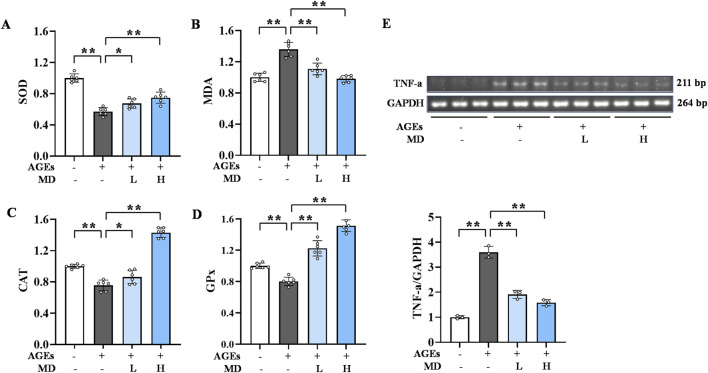
Inhibitory effect of MD on oxidative stress and inflammation induced by AGEs in osteoblasts. **(A)** The levels of SOD in osteoblasts (n = 6); **(B)** The levels of MDA in osteoblasts (n = 6); **(C)** The levels of CAT in osteoblasts (n = 6); **(D)** The levels of GPx in osteoblasts (n = 6); **(E)** The mRNA expression level of TNF-α in osteoblasts (n = 3). *P < 0.05; **P < 0.01.

The formation and action of AGEs can trigger oxidative stress, leading to an imbalance in cellular redox homeostasis (Moldogazieva et al., 2019). Figures 2A–D illustrates that the levels of SOD, CAT, and GPx were significantly decreased (*P* < 0.01), whereas MDA levels were notably increased (*P* < 0.01) in the AGEs group compared to the normal group. Conversely, the MD treatment groups exhibited significantly elevated levels of SOD, CAT, and GPx (*P* < 0.01) and markedly decreased levels of MDA (*P* < 0.01) when compared to the AGEs group. These results suggest that MD can elevate the intracellular levels of SOD, CAT, and GPx, while reducing MDA levels, thereby inhibiting AGEs-induced oxidative stress in osteoblasts.

### 3.4 Inhibition of AGEs-induced inflammatory response in osteoblasts by MD

AGEs promote the inflammatory response in cells, increasing the release of inflammatory factors (Deo et al., 2020). Semi-quantitative PCR was used to detect TNF-α levels (Figure 2E). Compared to the normal group, the AGEs group showed significantly elevated TNF-α mRNA expression in osteoblasts (*P* < .01). However, compared to the AGEs group, the MD treatment groups had significantly lower TNF-α mRNA levels (*P* < .01). These results suggest that MD can reduce TNF-α levels in osteoblasts, thereby inhibiting the inflammatory response induced by AGEs.

### 3.5 Downregulation of RAGE protein expression by MD

**FIGURE 3:**
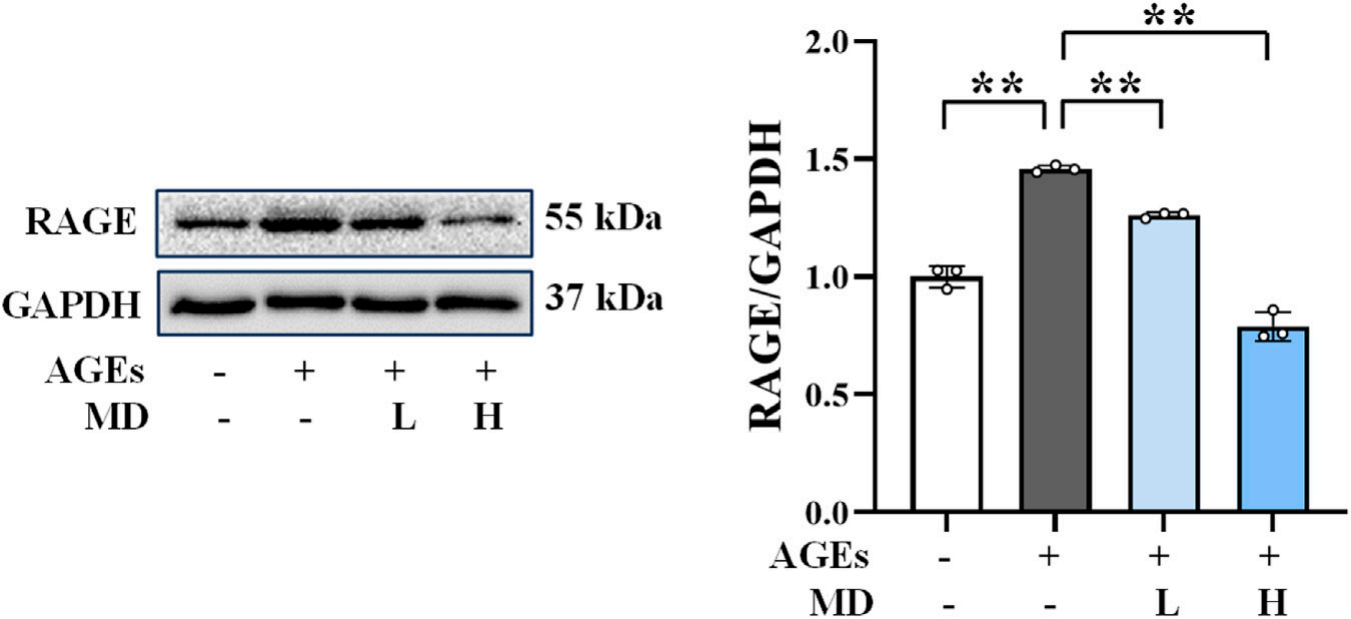
Western blotting results of RAGE in osteoblasts (n = 3). **P < 0.01.

Western blot analysis was conducted to detect RAGE expression to verify whether the protective effect of MD on AGEs-induced osteoblast damage is related to the RAGE pathway. As depicted in Figure 3, the MD treatment groups demonstrated a significant reduction in RAGE protein expression (*P* < 0.01) compared to the AGEs group. These results align with the findings observed for ALP, OCN, RUNX2, SOD, CAT, GPx, MDA, and TNF-α. These results suggest that the protective effect of MD on AGEs-induced osteoblast damage may be associated with the inhibition of RAGE activation.

### 3.6 Analysis of chemical components in MD using cell membrane chromatography

**FIGURE 4:**
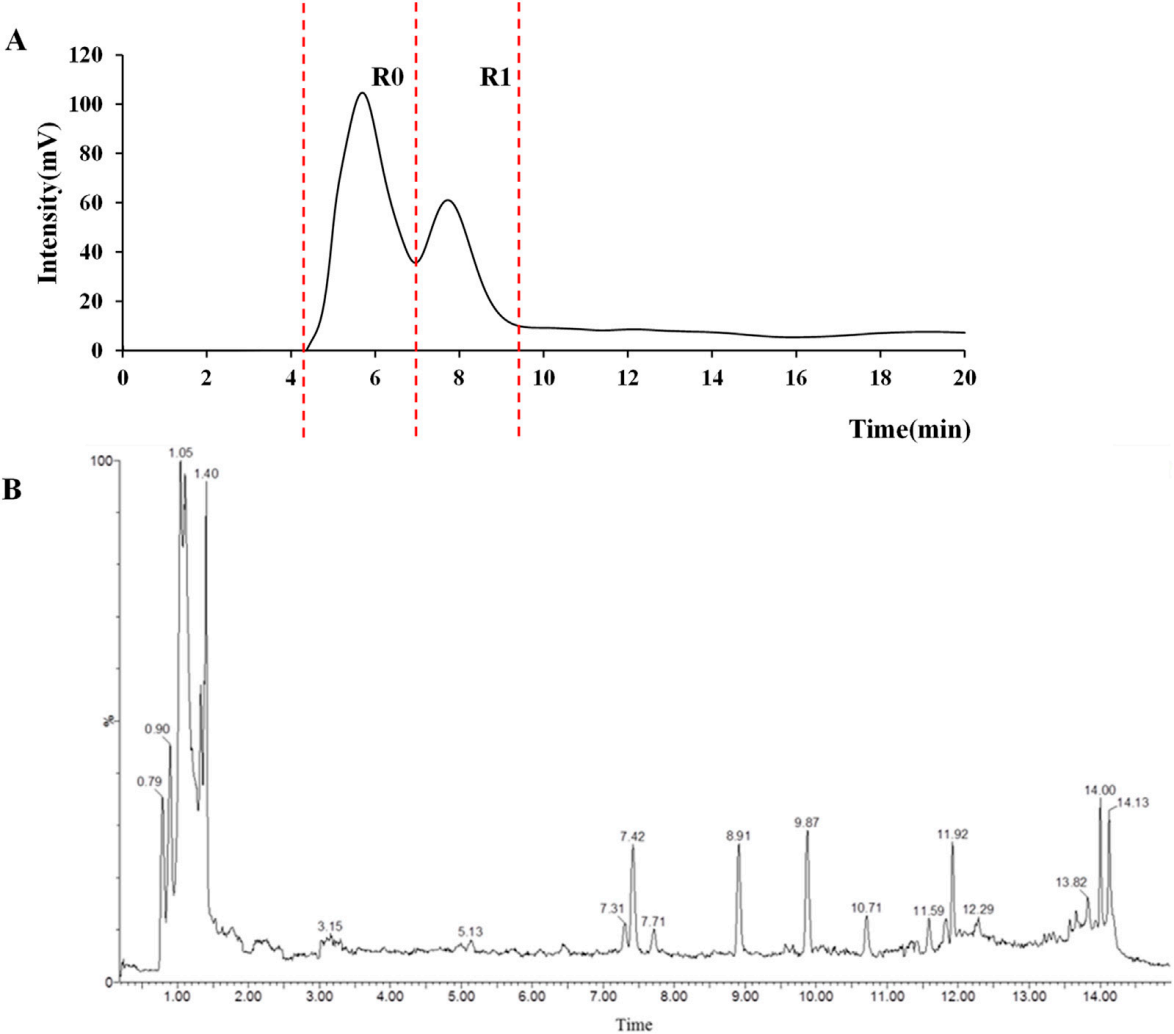
Chromatograms of MD extract using the osteoblast CMC-HPLC–TOF-MS method. **(A)** HPLC chromatogram of cell membrane chromatography; **(B)** Total ion chromatogram of retained components in negative ion mode from cell membrane chromatography.

The CMC method was employed to screen for potential active compounds with various structures in osteoblasts (Wang N et al., 2017). Figure 4A shows the screening results of cell membrane chromatography, where R0 represents the non-retained fraction of the HPLC chromatogram, and R1 represents the retained fraction. The retained components were identified using HPLC-TOF-MS, with the total ion chromatogram in negative ion mode shown in Figure 4B. A total of 20 potential active compounds were identified, including 14 flavonoids, 3 organic acids, 2 phenylpropanoids, 2 terpenoids, and 1 alkaloid. The structures of 20 compounds were confirmed, and their fragment information is listed in Table 2. Seven active components (gallic acid, protocatechuic acid, orientin, luteolin, isovitexin, rutin, and chlorogenic acid) were identified by comparison with standards, while the remaining active components were determined by comparison with literature.

**TABLE 2:** Analysis of MD components retained in AGEs modeled osteoblast cell membrane chromatography.

No.	tR (min)	Measured value (m/z)	Theoretical value (m/z)	Error (ppm)	Molecular formula	Major fragment ions (m/z)	name	Structure type	Category
1	1.22	353.0841	353.0873	−9.06	C_16_H_18_O_9_	179.0553、173.0491	Chlorogenic acid	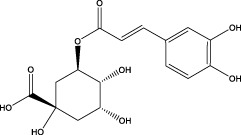	Organic acid
2	1.39	169.0149	169.0137	7.10	C_7_H_6_O_5_	125.0231、97.0332	Gallic acid	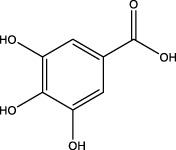	Organic acid
3	2.20	315.0154	315.0141	4.13	C_15_H_8_O_8_	299.2317、242.3152	3-O-methylellagic acid	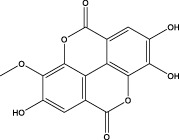	Organic acid
4	3.00	153.0195	153.0188	4.57	C_7_H_6_O_4_	136.0629、109.2862、91.0858	Protocatechuic acid	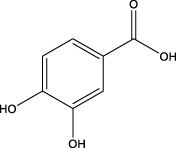	Organic acid
5	3.96	353.0867	353.0873	−1.70	C_16_H_18_O_9_	191.0380、213.3510、148.4750	Scopolin	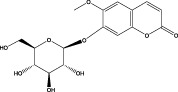	Phenylpropanoid
6	5.15	385.1850	385.1862	−3.12	C_19_H_30_O_8_	153.1451、207.6976、223.1254	1-enyl]cyclohex-2-en-1-one	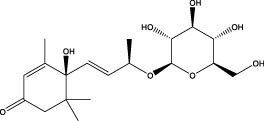	Terpenes
7	5.50	435.1085	435.1080	1.15	C_24_H_20_O_8_	341.3110	epicatechin-[8,7-e]-4β-(4-hydroxyphenyl)-3,4-dyhydroxyl-2(3H)-pyranone	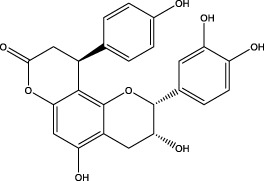	Flavone
8	6.09	447.0930	447.0927	0.67	C_21_H_20_O_11_	117.6527、173.5855	Quercitrin	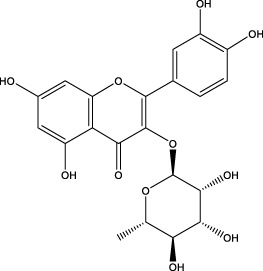	Flavone
9	6.46	447.0923	447.0927	−0.89	C_21_H_20_O_11_	327.0374、297.0730	Orientin	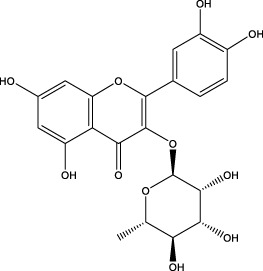	Flavone
10	6.47	447.0900	447.0927	−6.04	C_21_H_20_O_11_	285.0438	Luteoloside	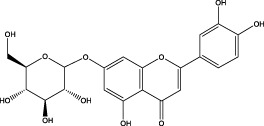	Flavone
11	7.30	609.1477	609.1456	3.45	C_27_H_30_O_16_	300.0248	Rutin	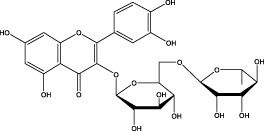	Flavone
12	7.42	431.0960	431.0978	−4.18	C_21_H_20_O_10_	268.0461、267.0597	Sophoricoside	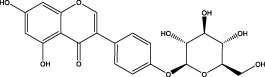	Flavone
13	7.42	431.0998	431.0978	4.64	C_21_H_20_O_10_	431.0975、323.0492、311.0571、283.0607、281.0437、269.0372	Isovitexin	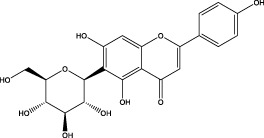	Flavone
14	7.79	611.1067	611.1037	4.91	C_29_H_24_O_15_	317.9065、302.0100	[6-[5,7-dihydroxy-2-(4-hydroxyphenyl)-4-oxochromen-3-yl]oxy-3,4,5-trihydroxyoxan-2-yl]methyl-3,4,5-trihydroxybenzoate	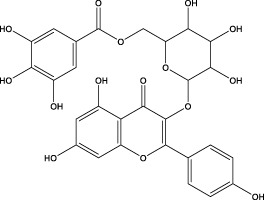	Flavone
15	7.82	463.0883	463.0877	1.30	C_21_H_20_O_12_	191.0333、151.0405	quercetin-3-O-β-D-glucopyranoside	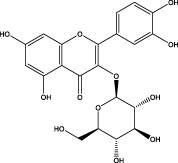	Flavone
16	7.82	463.0863	463.0877	−3.02	C_21_H_20_O_12_	301.0211、300.0276、271.0315、255.0391	Hyperin	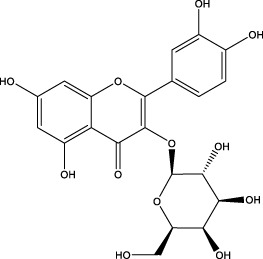	Flavone
17	8.54	593.1559	593.1506	8.94	C_27_H_30_O_15_	285.0433	Glucosylvitexin	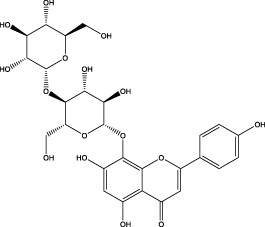	Flavone
18	9.15	447.0966	447.0927	8.72	C_21_H_20_O_11_	227.0369/255.0241、	kaempferol-O-glucoside	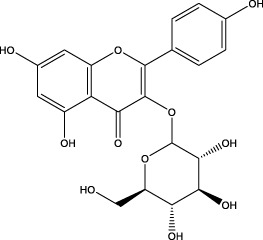	Flavone
19	13.15	582.2683	582.2604	13.57	C_34_H_37_N_3_O_6_	316.9684	Tricoumaroyl spermidine	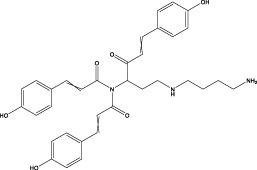	Alkaloid
20	14.10	457.0758	457.0771	−2.84	C_22_H_18_O_11_	125.8531	Epigallocatechin gallate	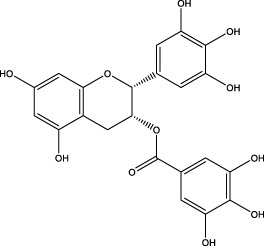	Flavone

Isovitexin showed a strong affinity for the osteoblast cell membrane among the screened compounds. Previous investigations have demonstrated that isovitexin possesses various pharmacological activities, such as hypoglycemic, anti-inflammatory, and antioxidant effects (Abdulai et al., 2021; He et al., 2016). Therefore, further studies on isovitexin were conducted.

In the previous study, we set up the HPLC fingerprint of MD (Supplementary Figure S1). According to the spectrum of the standard, we checked the sample peak of MD, and the components determined in MD are shown in Supplementary Table S1. Among them, the content of isovitexin is the highest, which is of great significance for the subsequent extraction, separation and application.

Relevant studies indicated that isovitexin can exert therapeutic effects on diabetes and related complications by alleviating apoptosis, regulating the hypothalamic-gonadal axis, targeting organs affected by persistent hyperglycemia, and inhibiting inflammation and oxidative stress (Abdulai et al., 2021). Pal et al. found that the bone formation-promoting effect of isovitexin in OVX mouse models is similar to teriparatide, suggesting its potential as a treatment for osteoporosis (Pal et al., 2022).

In view of the beneficial effect of isovitexin on diabetes and bone formation and its high content in MD, it is hypothesized that isovitexin is an active component of MD, potentially protecting against AGEs-induced osteoblast damage by activating RAGE.

### 3.7 Effect of isovitexin on the viability of AGEs-induced osteoblasts

**FIGURE 5:**
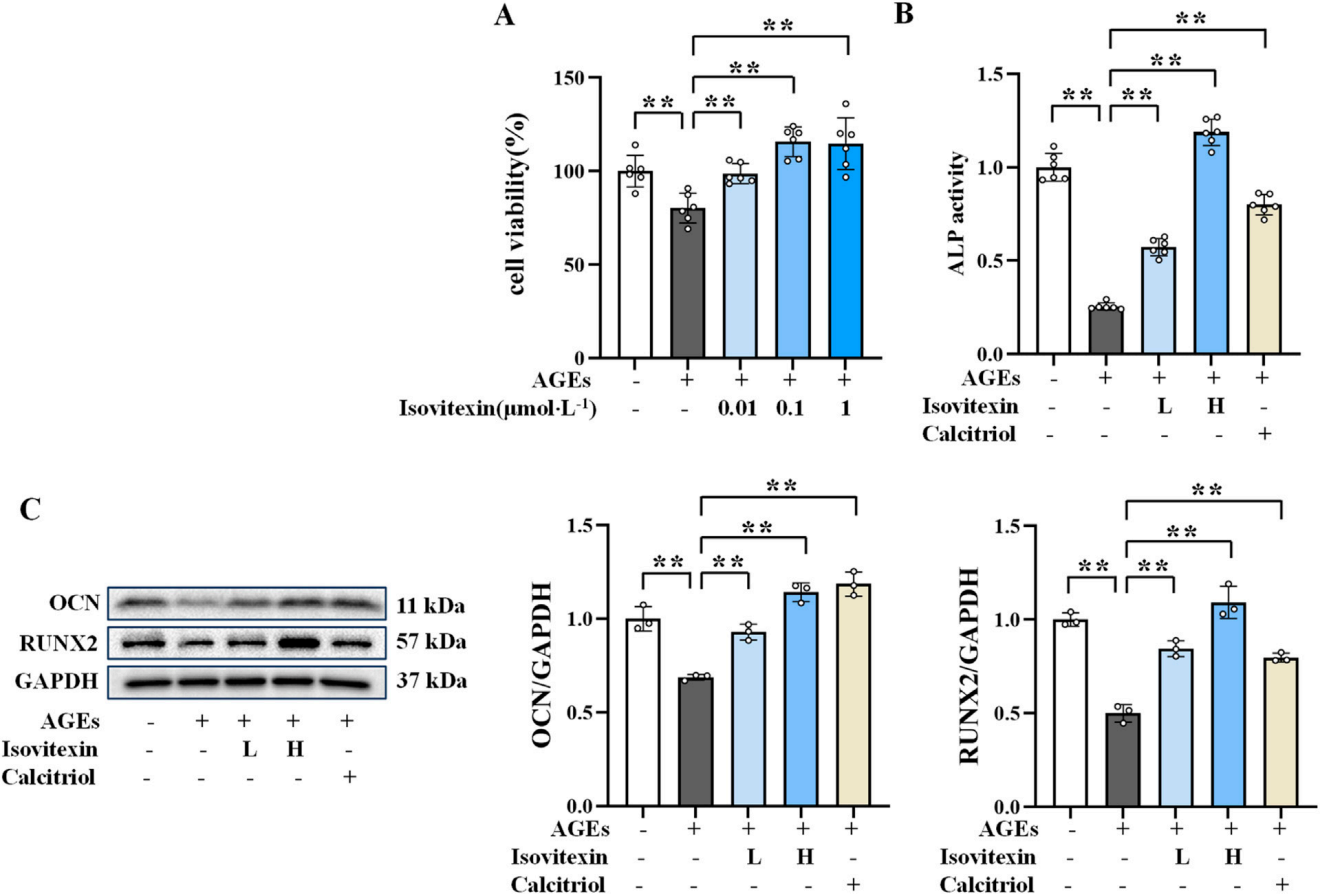
Protective effect of isovitexin on AGEs-induced osteoblasts. **(A)** Effect of isovitexin on the survival rate of osteoblasts (n = 6); **(B)** ALP activity in osteoblasts (n = 6); **(C)** Western blotting results of OCN and RUNX2 in osteoblasts (n = 3). **P < 0.01.

Given that isovitexin may be an active component of MD in treating AGEs-induced osteoblast damage, its effects were further investigated. The experimental results (Figure 5A) indicated that isovitexin at concentrations of 0.1, 1, and 10 μmol/L significantly promoted osteoblast proliferation (*P* < 01). No significant difference in efficacy was observed between the concentrations of 1 μmol/L and 10 μmol/L. Therefore, 0.1 and 1 μmol/L were chosen as the low and high doses for isovitexin treatment in this experiment.

### 3.8 Protective effect of isovitexin on AGEs-induced osteoblasts

Pal et al. (2022) found that isovitexin improves bone quality in OVX mice by upregulating the expression of osteogenic genes (Runx2, BMP-2, and collagen type I). ALP activity and the protein expression levels of OCN and RUNX2 in osteoblasts were measured to evaluate the bone formation-promoting effect of isovitexin. As shown in Figures 5B, C, compared to the AGEs group, the isovitexin treatment groups exhibited significantly increased ALP activity and higher expression levels of OCN and RUNX2 proteins (*P* < 0.01). These results indicate that 0.1 and 1 μmol/L doses of isovitexin enhance ALP activity and increase the expression levels of OCN and RUNX2 proteins, thereby protecting against AGEs-induced osteoblast damage.

### 3.9 Inhibition of AGEs-induced oxidative stress in osteoblasts by isovitexin

**FIGURE 6:**
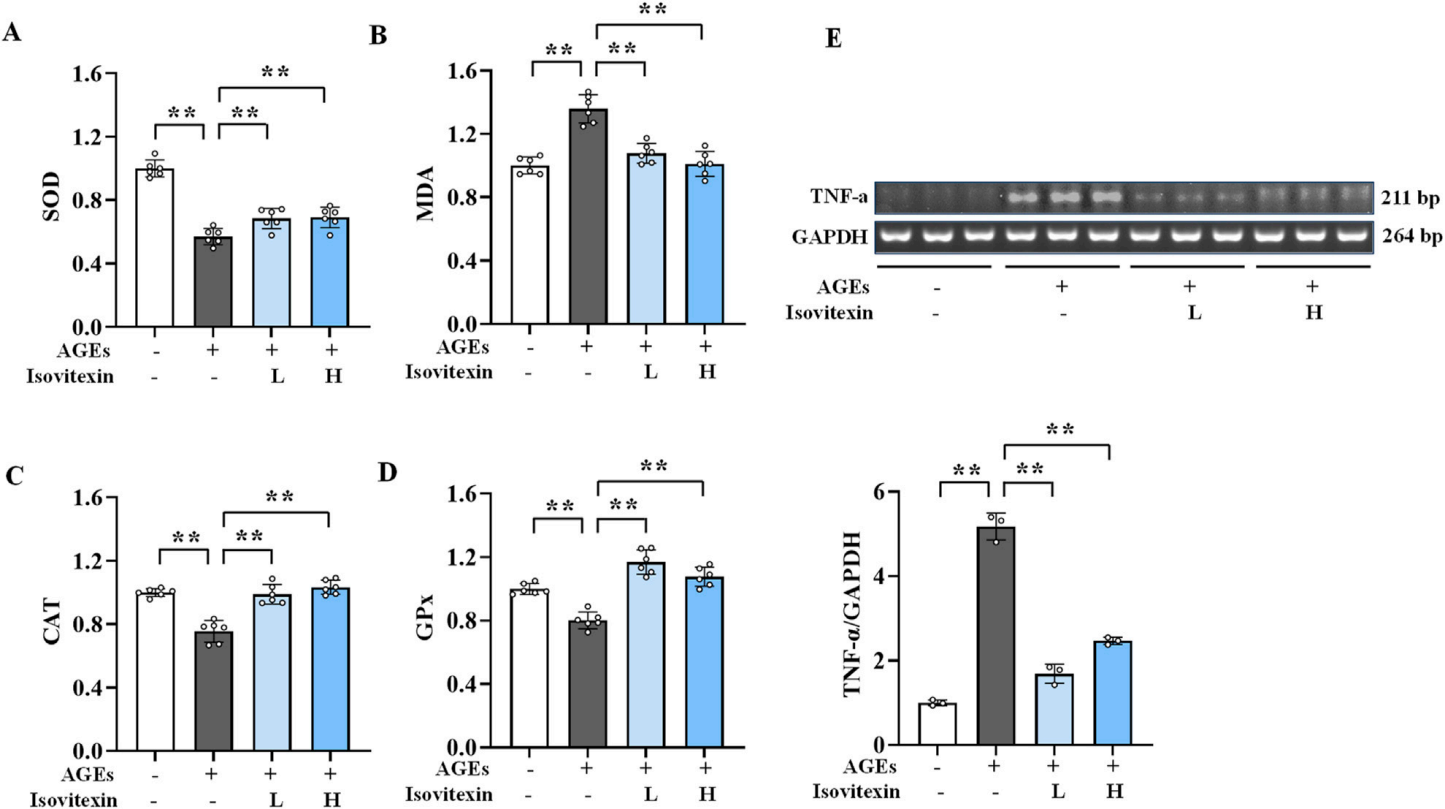
Inhibitory effect of isovitexin on oxidative stress and inflammation induced by AGEs in osteoblasts. **(A)** The levels of SOD in osteoblasts (n = 6); **(B)** The levels of MDA in osteoblasts (n = 6); **(C)** The levels of CAT in osteoblasts (n = 6); **(D)** The levels of GPx in osteoblasts (n = 6); **(E)** The mRNA expression level of TNF-α in osteoblasts (n = 3). **P < 0.01.

The results (Figures 6A–D) showed that compared to the normal group, the levels of SOD, CAT, and GPx were significantly reduced *(P* < 0.01), while the MDA level was significantly increased (*P* < 0.01) in the AGEs group. Conversely, the low and high doses of isovitexin significantly elevated the levels of CAT and GPx (*P* < 0.01) and reduced the level of MDA (*P* < 0.01) in osteoblasts compared to the AGEs group. These results indicate that isovitexin can elevate the intracellular levels of SOD, CAT, and GPx while reducing the level of MDA, thereby inhibiting AGEs-induced oxidative stress in osteoblasts.

### 3.10 Inhibition of AGEs-induced inflammatory response in osteoblasts by isovitexin

Semi-quantitative PCR was used to detect TNF-α levels (Figure 6E). The results showed that, compared to the normal group, the AGEs group had significantly elevated TNF-α mRNA expression in osteoblasts (*P* < 0.01). However, compared to the AGEs group, the isovitexin treatment groups had significantly lower TNF-α mRNA levels (*P* < 0.01). These findings indicate that isovitexin can downregulate the expression of TNF-α mRNA, thereby inhibiting the inflammatory response induced by AGEs in osteoblasts.

### 3.11 Downregulation of RAGE protein expression by isovitexin

**FIGURE 7:**
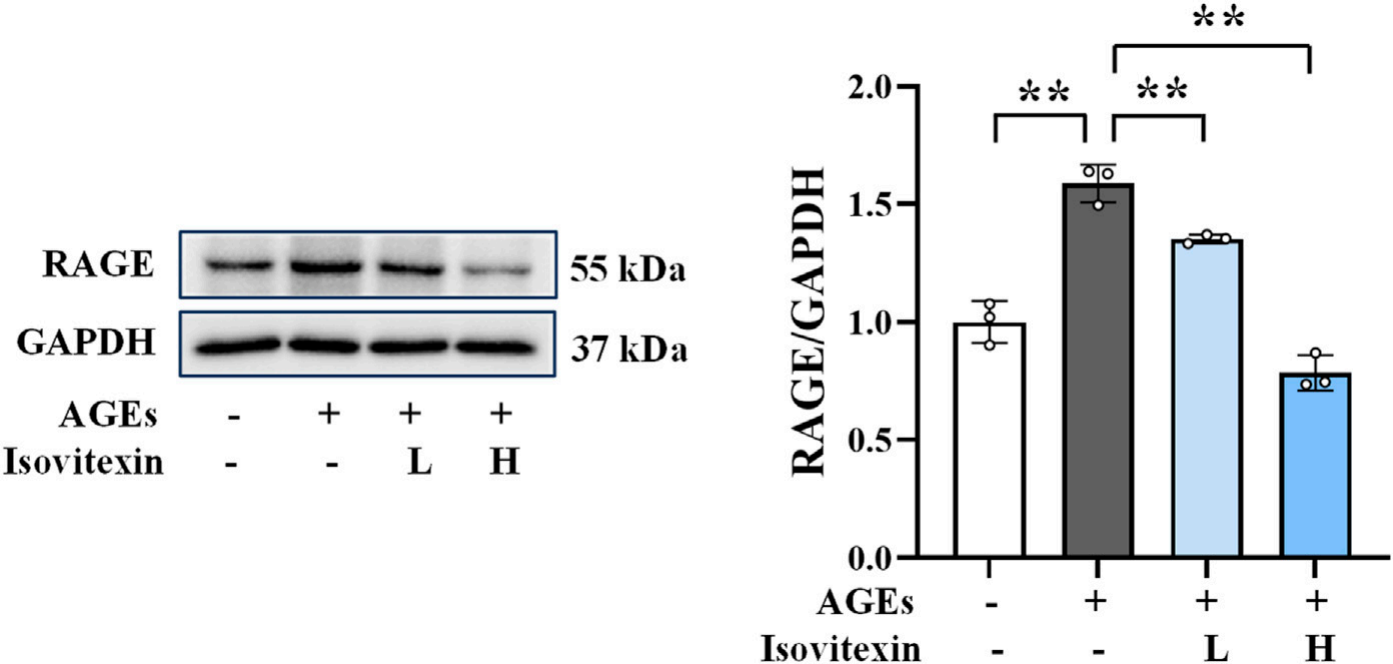
Western blotting results of RAGE in osteoblasts (n = 3). **P < 0.01.

Western blot analysis was performed to assess the levels of RAGE expression (Figure 7). The findings demonstrated a significant increase in RAGE expression in the AGEs group as opposed to the control group (*P* < 0.01). Conversely, the isovitexin treatment groups displayed significantly decreased RAGE expression levels (*P* < 0.01) compared to the AGEs group. These results suggest that isovitexin may exert its protective effect against AGEs-induced osteoblast damage by inhibiting the activation of RAGE, consistent with the effects observed for MD.

## 4 Discussion

CMC is a biomimetic chromatographic method based on the specific interaction between ligands and receptors. It mimics the *in vivo* interaction of drugs with membrane receptors in an *in vitro* chromatographic process, combining the biological activity of cell membranes with the separation characteristics of chromatography. In recent years, CMC has been widely used for screening active components in TCM for anti-tumor, anti-allergy, cardiovascular disease, anti-osteoporosis, anti-inflammatory, analgesic, antiviral, and anti-prostatic hyperplasia activities (Han et al., 2018; Ma et al., 2021). Our team previously established a mature CMC screening method using osteoblast cell membranes. However, it has been reported that receptor states in cells can undergo significant changes under pathological conditions, affecting the binding of chemical components to receptors. Therefore, screening for pharmacologically active components in TCM against diseases like DOP using cell membranes derived from pathological tissues is more scientifically sound.

This study established a CMC column using AGEs-induced pathological osteoblast membranes. HPLC-MS was utilized to identify, separate, and characterize the affinity active components in MD that interact with AGEs-induced pathological osteoblast membranes. A total of 20 retained components were screened, including 14 flavonoids, 3 organic acids, 2 phenylpropanoids, 2 terpenoids, and 1 alkaloid.

The production and deposition of AGEs in bone tissue are major contributors to diabetic osteoporosis (Cavati et al., 2023). A large amount of AGEs binding to RAGE on osteoblast membranes leads to osteoblast dysfunction. Additionally, RAGE activation enhances inflammatory and oxidative stress responses, further reducing the viability and function of osteoblasts and promoting their dysfunction (Ott et al., 2014; Jiang et al., 2022). AGEs have been shown to induce TNF-α production in osteoblasts. TNF-α is an inflammatory factor that plays a central role in immune homeostasis and inflammation and can induce various effects, such as necrosis and apoptosis. AGEs can promote bone resorption and inhibit bone formation by increasing TNF-α expression and secretion, directly or indirectly affecting osteoblasts and osteoclasts (Wang et al., 2017a; Kuang et al., 2017; Ma et al., 2017). Moreover, oxidative stress plays a crucial role in bone metabolism and is a major cause of osteoporosis, as it reduces osteoblast activity and number, accelerating bone loss (Li et al., 2021). Behera et al. found that regulating the miR-150-FNDC5/pyroptosis axis by inhibiting oxidative stress can improve diabetic osteoporosis (Behera et al., 2022). Therefore, enhancing antioxidant capacity and inhibiting inflammatory responses are beneficial for preventing and treating DOP.

This study found that MD and its active component, isovitexin, have protective effects against AGEs-induced osteoblast damage. They can mitigate the inhibitory effect of AGEs on osteoblast proliferation, upregulate OCN and RUNX2 protein expression, enhance ALP activity, increase osteoblast viability, downregulate RAGE protein expression, inhibit RAGE activation, elevate intracellular levels of SOD, CAT, and GPx, reduce MDA levels, inhibit oxidative stress, and decrease the mRNA levels of the inflammatory factor TNF-α. Lee et al. (2020) found that coumarins maintain bone homeostasis and treat DOP by inhibiting AGEs-RAGE activation. Chen et al. (2014) discovered that AGEs-induced upregulation of TNF-α levels in rabbit chondrocytes can be reversed by inhibiting RAGE activation. Franke et al. (2011) found that AGEs-induced osteoblast damage increases the expression of RAGE and TNF-α while downregulating osteogenic markers OPG, Col1, OCN, and ALP, resulting in decreased osteogenic activity. Liu et al. (2018) reported that mulberry leaf extract could regulate the PTH/VDR/CaBP and AGEs/RAGE/Nox4/NF-κB signaling pathways, upregulate OCN and OPG expression, downregulate AGEs, RAGE, Nox4, NF-κB, and RANKL expression, enhance bone protection, increase SOD and TAC levels, and reduce IL-6 and MDA levels, thereby inhibiting oxidative stress and inflammatory responses. These findings align with the results of the present study.

Osteoclast is an important cell in the skeletal system, which is responsible for bone absorption and reconstruction. In the pathological process of osteoporosis, the activity of osteoclasts is often enhanced, resulting in bone mass reduction and bone mineral density reduction (Wang et al., 2017b; He et al., 2019). Therefore, inhibiting the activity of osteoclasts and promoting the activity of osteoblasts are also one of the important strategies for the treatment of osteoporosis. Xu et al. found that 50% ethanol extract of Rehmannia glutinosa could inhibit RANKL induced osteoclast differentiation and reduce the number of osteoclasts (Xu, 2023). The research on the osteogenic and osteoclastic effects of MD is not in-depth at present, but its application prospect as a traditional Chinese medicine in the treatment of osteoporosis is still worthy of attention. Currently, research on MD in the field of DOP is relatively limited. This study demonstrates that MD can protect against AGEs-induced osteoblast damage by inhibiting the activation of the RAGE pathway. Isovitexin was identified as an active component with affinity for osteoblast membranes and ameliorative effects on AGEs-mediated osteoblast damage. In summary, MD and its active component, isovitexin, exhibit protective effects in an AGEs-induced osteoblast damage model, providing a basis for the development of drugs for DOP.

## Data Availability

The original contributions presented in the study are included in the article/Supplementary Material, further inquiries can be directed to the corresponding author.
