# Supplementary material for: Screening of active components of melastoma dodecandrum lour. against diabetic osteoporosis using cell membrane chromatography-mass spectrometry
Source: Front Pharmacol. 2024 Oct 25;15:1450154. doi: 10.3389/fphar.2024.1450154 (PMC11543422; doi:10.3389/fphar.2024.1450154)
Supplement: Supplementary file 3 [file DataSheet1.docx]

 **Supplementary Figure 1** Fingerprint of MD.1 gallic acid, 2 protocatechuic acid, 3 ethyl gallate, 4 orientin, 5 isovitexin, 6 rutin, 7 isoquercitrin, 8 3-O-methylellagic acid, 9 afzelin.

**Supplementary Table 1** Components content of MD.

| No. | name | t_R_(min) | content (µg) |
| --- | --- | --- | --- |
| 1 | gallic acid | 7.30 | 1.487 |
| 2 | protocatechuic acid | 13.12 | 0.276 |
| 3 | ethyl gallate | 27.94 | 0.479 |
| 4 | orientin | 33.95 | 0.469 |
| 5 | isovitexin | 43.06 | 4.020 |
| 6 | rutin | 48.04 | 0.518 |
| 7 | isoquercitrin | 52.19 | 1.234 |
| 8 | 3-O-methylellagic acid | 64.04 | 0.087 |
| 9 | afzelin | 74.24 | 0.043 |
